# Bone Diseases in Patients with Chronic Liver Disease

**DOI:** 10.3390/ijms20174270

**Published:** 2019-08-31

**Authors:** Hae Min Jeong, Dong Joon Kim

**Affiliations:** 1 Department of Internal Medicine, Hallym University Chuncheon Sacred Heart Hospital, Chuncheon, Gangwon-do 24253, Korea; 2 Institute for Liver and Digestive Diseases, Hallym University, Chuncheon, Gangwon-do 24253, Korea; 3 Department of Internal Medicine, Hallym University College of Medicine, Chuncheon, Gangwon-do 24252, Korea

**Keywords:** osteoporosis, liver disease, liver cirrhosis, biliary cholangitis, liver transplantation, tenofovir disoproxil fumarate, sarcopenia, dysbiosis

## Abstract

Osteoporosis is a frequently observed complication in patients with chronic liver disease, particularly liver cirrhosis and cholestatic liver diseases. In addition, osteoporosis is critical in patients receiving a liver transplant. Nevertheless, few studies have evaluated bone diseases in patients with more frequently observed chronic liver disease, such as chronic viral hepatitis, nonalcoholic fatty liver disease and alcoholic liver disease. Osteoporosis is a disease caused by an imbalance in the activities of osteoblasts and osteoclasts. Over the last few decades, many advances have improved our knowledge of the pathogenesis of osteoporosis. Importantly, activated immune cells affect the progression of osteoporosis, and chronic inflammation may exert an additional effect on the existing pathophysiology of osteoporosis. The microbiota of the intestinal tract may also affect the progression of bone loss in patients with chronic liver disease. Recently, studies regarding the effects of chronic inflammation on dysbiosis in bone diseases have been conducted. However, mechanisms underlying osteoporosis in patients with chronic liver disease are complex and precise mechanisms remain unknown. The following special considerations in patients with chronic liver disease are reviewed: bone diseases in patients who underwent a liver transplant, the association between chronic hepatitis B virus infection treatment and bone diseases, the association between sarcopenia and bone diseases in patients with chronic liver disease, and the association between chronic liver disease and avascular necrosis of the hip. Few guidelines are currently available for the management of low bone mineral density or bone diseases in patients with chronic liver disease. Due to increased life expectancy and therapeutic advances in chronic liver disease, the importance of managing osteoporosis and other bone diseases in patients with chronic liver disease is expected to increase. Consequently, specific guidelines need to be established in the near future.

## 1. Introduction

Osteoporosis, which results in a high risk of fragility fractures, is a frequently observed complication in patients with chronic liver disease, especially in liver cirrhosis, cholestatic liver diseases and hemochromatosis [1]. In addition, the problem is critical in patients who undergo a liver transplant when bone loss is accelerated, leading to a greater incidence of fractures during the period immediately after transplantation [2]. Nevertheless, few studies have evaluated bone diseases in patients with more frequently observed chronic liver disease, such as chronic viral hepatitis, non-alcoholic fatty liver disease and alcoholic liver disease [3,4,5,6,7,8,9].

The detection of osteoporosis in patients with chronic liver disease requires a high index of suspicion, as approximately one-third of vertebral fractures are asymptomatic [4]. In contrast, femoral neck fractures are uncommon in patients with liver cirrhosis as they occur approximately a decade later than vertebral fractures, which is beyond the life expectancy of most patients with cirrhosis [4].

In the past, bone diseases in patients with chronic liver disease were described using the term “hepatic osteodystrophy” [4,5,8,10,11]. However, this designation includes osteomalacia caused by impaired bone mineralization, which is rare and has been reported in isolated patients with advanced primary biliary cholangitis (PBC) and severe intestinal malabsorption in geographic areas with limited sunlight exposure [12]. Currently, the most frequently diagnosed bone diseases in patients with chronic liver disease are osteoporosis and osteopenia [13].

In the last few decades, many developments have improved our knowledge of the pathogenic mechanisms and management of osteoporosis [3,14,15]. However, there is no consensus on the global guidelines for the diagnosis and treatment of bone diseases as a complication of chronic liver disease and many issues remain to be solved in patients with chronic liver disease [16]. Therefore, additional studies should be conducted on the topic of bone diseases in patients with chronic liver disease in the future. In this review, we describe the definitions of related terms, guidelines for bone diseases and chronic liver disease, current knowledge of pathogenesis, and finally focus on the management of bone diseases in patients with chronic liver disease.

## 2. Definitions of Related Terms

First, we define related terms that are used in this review.

Osteoporosis is a systemic bone disease characterized by a low bone mineral density (BMD) and susceptibility to fracture [17]. According to the World Health Organization (WHO) definition, osteoporosis is diagnosed when the bone density is less than −2.5 standard deviations below the peak value obtained from normal adults and adjusted for gender (T-score ≤ −2.5 in postmenopausal women, or Z-score ≤ −2 in men less than 50 years old and premenopausal women) [1,10,15,18]. The evaluated bone should be free from other systemic problems, including osteomalacia, or local abnormalities, such as osteophytes, extra-skeletal calcifications, or deformities due to previous fractures [9,15,19]. Osteopenia is defined as a T-score between −1 and −2.5 [1,15].

Studies investigating the association between bone diseases and chronic liver disease have mainly focused on cholestatic liver diseases [20]. Since patients with liver cirrhosis and other liver diseases also exhibit bone diseases, in this review, chronic liver disease encompasses, but is not limited to, liver cirrhosis and cholestatic liver diseases. Severe cholestatic liver diseases are defined as the presence of a serum bilirubin level greater than three times the upper limit of normal for more than six months [9]. PBC and primary sclerosing cholangitis (PSC) are typically included in cholestatic liver disease.

Sarcopenia is a potentially lethal extra-hepatic manifestation in patients with chronic liver disease, particularly in patients with cirrhosis, but its definition remains unclear [21]. Recently, Carey et al. identified and defined the skeletal muscle index (SMI) (SMI cutoffs of 50 cm^2^/m^2^ for men and 39 cm^2^/m^2^ for women) [22]. This cutoff value determines the numerical definition of sarcopenia by showing a clear survival difference in patients with end-stage liver disease.

Avascular necrosis, also known as osteonecrosis of the femoral head, is a special bone disease that is the final common pathway of a series of derangements which result in a decrease in blood flow to the femoral head, leading to cellular death, fracture and collapse of the articular surface [23].

The gut microbiota has recently been identified as an important contributor to the pathophysiology of many intestinal and extra-intestinal diseases [24,25,26]. The gut microbiota may affect bone diseases in patients with chronic liver disease. For example, an intestinal infection with pathogenic bacteria induces bone loss in male mice [27]. Thus, we will discuss the association between bone diseases in patients with chronic liver disease and the gut microbiota. The microbiota is the community of microbial species that live in a particular environment; the microbiome is all of the genomes of these microbial cells that constitute the microbiota [28]. Dysbiosis refers to the breakdown of the normal balance between the normal flora and other bacteria [29].

## 3. Guidelines for Bone Diseases in Patients with Chronic Liver Disease

Osteoporosis or osteopenia is a common complication in patients with cirrhosis. The prevalence of osteoporosis in patients with cirrhosis is approximately 12–55%, which is higher than in healthy people [6,18]. Notably, up to 40% of patients with chronic liver disease may experience a fracture [3,30]. Nevertheless, prospective studies or interventional studies that specifically focus on bone diseases in patients with chronic liver disease are sparse. Primary osteoporosis should be distinguished from forms of secondary osteoporosis [1]. One of the representative secondary causes is chronic liver disease [31,32].

The guidelines of most osteoporosis societies briefly describe the causes of fractures and osteoporosis in patients with chronic liver disease as nutritional deficiencies caused by liver disease [1,15]. Because a thorough review of the guidelines of osteoporosis societies is beyond the scope of this article, the guidelines of liver disease societies will be reviewed (Table 1) [2,9,20,33,34,35,36,37,38,39,40,41].

To date, only a limited set of guidelines has been introduced. The European Association for the Study of the Liver (EASL) introduced nutrition guidelines for patients with chronic liver disease in 2019 and has provided a new reference to bone diseases in patients with chronic liver disease [41]. The prevalence of osteoporosis in patients with chronic liver disease was reported to be approximately 30%. In addition, its prevalence was higher in patients with cholestatic liver diseases, and the prevalence of fractures was reported to be 7–35% [41]. In the EASL guidelines, screening, diagnosis, and nutritional support for bone diseases in patients with chronic liver disease have been mentioned, and several options for therapeutic agents have been proposed [41].

The American Association for the Study of Liver Disease (AASLD) updated the guidelines for PBC in 2018 and provided recommendations for the diagnosis and management of osteoporosis and osteopenia in patients with chronic liver disease [40]. Patients with PBC have a significantly greater risk of osteopenia and osteoporosis than age-matched and sex-matched controls [40]. Although the AASLD guidelines suggested diagnoses and management strategies for osteoporosis and osteopenia in patients with chronic liver disease, chronic liver disease is only defined as a secondary risk factor for osteoporosis or fracture.

The estimated prevalence of fragility fractures is approximately 10–15% in patients waiting for solid organ transplants (kidney, heart, liver and lung) [1]. In particular, in the case of liver transplantation (LT), patients already had a bone disease because they were diagnosed with a chronic liver disease prior to transplantation [42]. In addition, the use of immunosuppressants after transplantation further exacerbates bone diseases [35]. LT itself is not thought to increase the risk of fracture. The AASLD LT guideline recommends that bone densitometry and measurements of vitamin D and calcium levels are performed prior to the LT [35]. This topic will be described in detail below as one of the special considerations.

## 4. Pathogenesis

Osteoporosis is a disease caused by an imbalance in the activities of osteoblasts and osteoclasts [3]. Importantly, activated immune cells affect the progression of osteoporosis, and chronic inflammation may exert an additional effect on the existing pathophysiology of osteoporosis [43]. Recently, the microbiota of the intestinal tract were postulated to affect the progression of bone loss in patients with chronic liver disease [27,29,44]. However, the mechanisms underlying osteoporosis in patients with chronic liver disease are complex and not completely understood [45].

In general, the mechanisms of osteoporosis are described as follow. Osteoblasts and osteoclasts play a major role in the progression of osteoporosis by regulating bone remodeling [3,46]. In normal physiology, bone remodeling is based on the balance of these activities, and both bone quantity and quality are maintained [3]. The site where bone remodeling occurs is called the bone remodeling unit (BRU), and approximately one million BRUs exist in adults [46,47,48]. This remodeling is tightly controlled by a variety of molecules and systemic proteins [7,30]. When the balance between osteoclasts and osteoblasts is disrupted and bone resorption occurs more rapidly than bone formation, the amount of bone decreases and osteoporosis progresses [3,49]. Osteoblasts generally undergo apoptosis after bone formation, but the remaining osteoblasts differentiate into osteocytes or bone-lining cells and survive for long periods [3,48]. This osteocyte, which is embedded in the matrix and is the mediator of bone remodeling in response to mechanical strain, has emerged as a major regulator of bone resorption by controlling the number of osteoclasts and bone formation by modifying osteoblast-induced bone mineralization [1,3,15,19].

In patients with chronic liver disease, the balance between osteoclasts and osteoblasts is affected by liver disease, resulting in BRU imbalance [46,50]. Most studies have observed more significant impairment in bone formation, suggesting that osteoporosis in patients with cirrhosis is a multi-factorial disease in which different mechanisms act together to reduce bone mass until skeletal fragility occurs [12]. However, different liver disease entities differ in their pathogenesis and hence the cause of the associated bone loss may also vary [30]. For example, while hemochromatosis and Wilson’s disease are associated with increased iron and copper load and hence may affect bone diseases by inhibiting osteoblasts [51,52,53]; viral hepatitis is associated with an activated immune response and the release of mostly resorption-activating cytokines (Table 2) [4,5,6,7,8,45,54,55,56].

### 4.1. Predominant Decrease in Bone Formation

**Figure 1:**
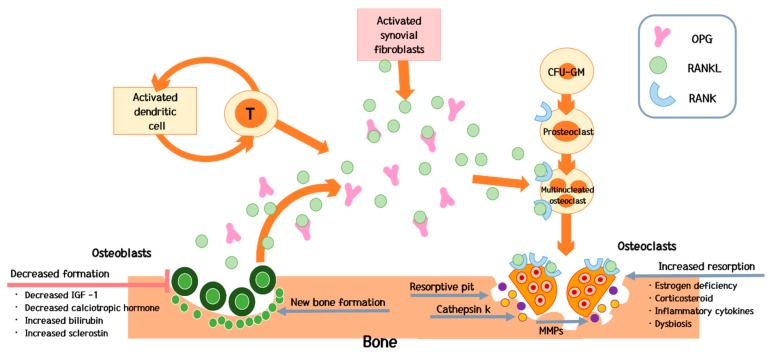
Pathophysiology of bone loss. Bone loss due to decreased bone formation is mostly a direct or indirect toxic effect on osteoblast differentiation and survival. In contrast, increased bone resorption resulting from the activation of osteoclasts is a cause of bone diseases in patients with chronic liver disease due to the effects of inflammation and hormones. In particular, activated immune cells such as T-lymphocytes and activated synovial fibroblasts are the primary source of receptor activator of nuclear factor κ-β ligand (RANKL), which activates osteoclasts. Activated osteoclasts secrete matrix metallopeptidases (MMP) and cathepsin K (Cat K), resulting in bone resorption. OPG: osteoprotegerin; RANK: receptor activator of nuclear factor κ-β.

Bone loss due to decreased bone formation is mostly a direct or indirect toxic effect on osteoblast differentiation and survival (Figure 1) [57]. In particular, this phenomenon has been observed in patients with cholestatic diseases [58]. For example, in patients with PBC and PSC, higher bilirubin levels exert a negative effect on osteoblasts [58]. In a study of patients with hemochromatosis, 25% had osteoporosis and 41% had osteopenia [30]. A lower BMD was reported in patients with higher iron loads and lower levels of markers of bone formation [59]. Although osteoblast proliferation, differentiation, and apoptosis are not as extensively studied as in osteoclasts, the current consensus is that chronic liver disease exerts a negative effect on osteoblast differentiation and proliferation [1].

**Figure 2:**
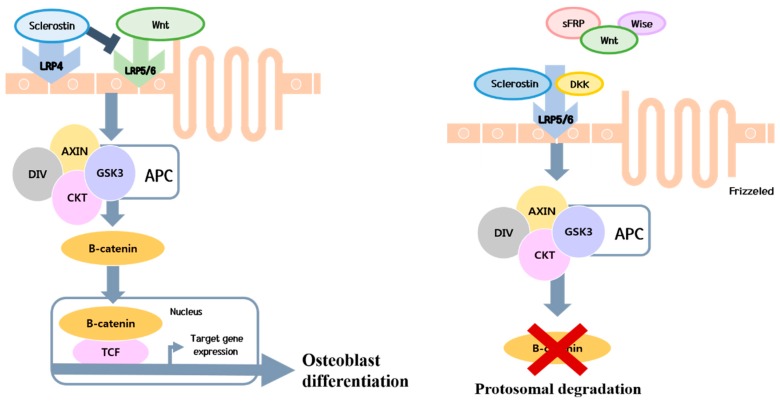
Effects of sclerostin on osteoblast differentiation. Sclerostin produced by osteocytes inhibits Wnt/β-catenin signaling during early bone diseases in patients with cholestatic diseases [3]. This soluble protein is produced by osteocytes that differentiated from osteoblasts and prevents Wnt from binding to low-density lipoprotein receptor-related proteins-5/6 (LRP5/6) transmembrane receptors. This blockade prevents Wnt signaling and osteoblast differentiation to inhibit bone formation. LRP: lipoprotein receptor-related proteins; GSK3: glycogen synthase kinase 3, DKK: Dickkopf; sFRP: secreted frizzled-related protein; APC: adenomatosis polyposis coli; TCF: T-cell specific transcription factor.

Sclerostin from osteocytes inhibits Wnt/β-catenin signaling during early bone disease in patients with cholestatic diseases (Figure 2) [5]. Sclerostin is a soluble protein secreted from osteocytes that differentiated from osteoblasts, which prevents Wnt from binding to low-density lipoprotein receptor-related proteins-5/6 transmembrane receptors [60]. This blockade prevents osteoblast differentiation to inhibit bone formation [60,61]. In a recent study, higher circulating sclerostin levels were observed in patients with advanced liver cirrhosis than in healthy controls or patients with early liver cirrhosis [62].

Osteoblast dysfunction may result from decreased levels of trophic factors such as insulin-like growth factor-1 (IGF-1) [5,63,64]. Bone is one of the major target organs for IGF-1, an anabolic hormone produced mainly by the liver upon growth hormone (GH) stimulation. [65]. IGF-1 is crucial to achieving normal longitudinal bone growth and mass during the postnatal period and plays a major role in bone growth and development [66]. IGF-1 reduces osteoblast apoptosis and promotes osteoblastogenesis by stabilizing β-catenin, and enhancing Wnt-dependent activity [67,68]. Thus, rapid decreases in serum IGF-1 levels after menopause might be partly involved in bone loss. Experimental data show that low doses of IGF-1 increase bone mass in cirrhotic rats [65,69,70]. Liu et al. also showed that if the serum level of IGF-1 at 1.5 SD below its peak was adopted as a cutoff point, it could identify women with low bone mass/osteoporosis with a sensitivity of 73% and specificity of 67% [71]. In advanced liver cirrhosis, IGF-1 serum levels decrease as a result of diminished hepatocellular biosynthetic function and progressive loss of GH receptors on hepatocytes [65,72]. Therefore, low serum IGF-1 levels could lead to bone diseases in patients with chronic liver disease. Hypogonadism which affects both osteoclasts and osteoblasts may be another important factor [73]. Some authors have hypothesized that low levels of sex hormones (estrogen or testosterone) increase the osteoclast life span and decrease the osteoblast life span, leading to a higher rate of bone resorption than new bone synthesis [67,73,74].

### 4.2. Predominant Increase in Bone Resorption

Parathyroid hormone (PTH) is a key regulator of calcium homeostasis [75]. At the level of the bone, it stimulates osteoblasts to form bone and osteoclasts to resorb bone [76]. Since osteoclasts do not express the PTH receptor, the effect of PTH on the osteoclasts is mediated by other molecules produced in response to PTH on osteoblasts [77], in particular receptor-activator of nuclear factor kappa ligand (RANKL) and osteoprotegerin system (OPG) [78].

The RANKL–RANK–OPG system is the key to understanding the mechanism of osteoporosis. RANKL and OPG are part of the tumor necrosis factor (TNF) superfamily, and are associated with inflammation and the immune system. Therefore, RANKL and OPG which are signaling through RANK have other functions beyond regulation of bone remodeling [79].

RANKL is a soluble protein secreted from osteoblasts that binds to a receptor on the surface of osteoclasts and then activates these cells [76]. RANKL is also produced by activated immune cells [80]. Many cells of the immune system express RANKL and RANK [81]. It is expressed by synovial cells and activated T cells in joints of patients with inflammatory arthritis [82]. Stimulation with recombinant RANKL *in vitro* enhances dendritic cell (DC) survival [83], adhesive properties and cytokine production, suggesting that RANKL stimulates antigen presentation to T cells [81]. T cells are essential mediators of bone loss in ovariectomized mice [81]. In a series of papers it was shown that athymic nude mice, which lack T cells, were protected from bone loss [84]. This finding indirectly suggests an effect of chronic inflammation on the bone, and cytokines, which are produced in the liver in patients with chronic liver disease, may contribute to osteoclast activation [43,82,85].

RANK is a homotrimeric transmembrane protein member of the TNF receptor superfamily [79]. It appears to be expressed in fewer tissues than RANKL at the protein level [79]. Macrophage-colony stimulating factor (M-CSF) induces RANK on osteoclast precursor cells and supports their proliferation [81]. M-CSF plays an important role in osteoclastogenesis [79], by binding with RANKL to promote RANK trimerization and activate intracellular signaling [81]. Kapur et al. confirmed that RANK is a receptor in osteoclastogenesis through transgenic mice by a deletion mutation of the gene that encodes RANK [86]. Thus RANKL/RANK signaling can regulate osteoclast formation, activation and survival in normal bone modeling and remodeling and in a variety of pathologic conditions characterized by increased bone turnover [79,83,85,86].

OPG is secreted by many cell types in addition to osteoblasts, including those in the heart, kidney, liver and spleen [79]. In the immune system, OPG is expressed in lymph nodes, B cells and DCs [87]. A recent study reports that B cells may be responsible for 64% of total bone marrow OPG production and B cell-deficient mice are known to be consistently osteoporotic, which is consistent with B cells being a major source of OPG in the bone marrow of normal mice [79]. The Wnt/β-catenin pathway also regulates osteoblastic bone formation and the commitment of mesenchymal cells to the osteoblast lineage [79]. This prevents RANKL from binding to receptors on osteoclasts [82]. Eventually OPG blocks activation of osteoclast by RANKL [79]. OPG’s osteoprotective role in humans has been supported in a study of homozygous partial deletions of *opg* gene in patients with juvenile Paget’s disease, an autosomal recessive disorder in which affected individuals have increased bone remodeling, osteopenia and fractures [79].

Taken together, the RANKL–RANK–OPG system is a key regulator of bone homeostasis in the setting of chronic inflammation [82]. Interleukin-6 (IL-6), Interleukin-1β (IL-1β) and TNFα are representative cytokines in chronic inflammation.

IL-6 has been thought of as a pro-inflammatory cytokine due to its elevation in numerous malignancies and inflammatory diseases [88]. The role of IL-6 in osteoclastogenesis was investigated [89,90,91]. IL-6 is produced by osteoblasts and either directly activates osteoclasts or stimulates osteoblasts to produce RANKL and thus indirectly activates osteoclasts [89,90,91]. A previous study revealed that IL-6 contributes to the defective osteogenesis of bone marrow stromal cell (BMSC) in the vertebral body of the osteoporotic mice and that the *in vivo* administration of an IL-6 neutralizing antibody can rescue this phenotype [92]. Indeed, the inhibition of the IL-6 receptor in mice blocked osteoclast-mediated bone resorption [93]. In the liver, IL-6 is up-regulated after injury and triggers the acute phase response and liver regeneration [94]. Since any type of liver injury is also associated with an attempt at regeneration, IL-6 is essentially up-regulated in patients with all types of liver disease [94,95].

IL-1β is a potent proinflammatory cytokine [96] involved in many important cellular functions, such as proliferation, activation and differentiation and is an important component of the innate immune response [97]. The IL-1 family of ligands includes 11 members among which IL-1β has emerged as the primary therapeutic target for an expanding number of inflammatory conditions [98]. IL-1β is a strong stimulator of bone resorption [99]. IL-1β up-regulates the production of RANKL and makes RANKL stimulate osteoclastogenesis [100]. A study revealed the role of IL-1 in physiological bone metabolism through the analyses of IL-1α deficient, IL-1β deficient and IL-1α/β double deficient mice that were housed under specific pathogen free conditions [101]. In this study, the femur mineral density, trabecular bone mass and cortical thickness significantly increased in all knockout (KO) mice compared with wild-type (WT) mice [101]. In addition, IL-1β increases prostaglandin synthesis in bone which displays a potent resorption stimulus [85]. After an inflammatory stimulus, prostaglandins, such as prostaglandin E2 (PGE2), may mediate the up-regulation of RANK by activating cell-surface receptors, thus regulating osteoclast differentiation, activation and survival [85].

TNF also plays a key role in the pathogenesis of inflammatory bone resorption [102]. TNF is an inflammatory cytokine important to immunity and inflammation. The importance of TNF in inflammation has been highlighted by the efficacy of anti-TNF antibodies or the administration of soluble TNF receptors to control disease activity in rheumatoid arthritis and other inflammatory conditions [103]. An established mechanism by which TNF promotes inflammatory bone resorption is the activation of osteoblasts and tissue stromal cells to express RANKL, the key factor that induces differentiation and function of osteoclasts [102]. Specially, TNFα promotes osteoclast formation [104]. During the early stages of osteoclastogenesis, TNFα increases the pool size of marrow osteoclast precursors by stimulating colony-stimulating factor-1 receptor gene expression, which is independent of the RANK pathway [104]. These osteoclast precursors then differentiate into mature osteoclasts in the presence of RANKL, and this process is accelerated by TNF. The role of TNF at this later stage of osteoclast differentiation is RANKL/RANK dependent [104].

In fact, rather than acting independently, IL-6, IL-1β and TNFα in combination activate osteoclasts through chronic inflammation, leading to bone resorption [91]. These cytokines share a common pathogenic mechanism of chronic inflammation. For example, if TNFα inhibition is independently involved in preventing bone loss, the sequestration of TNFα by the monoclonal antibody infliximab would be expected to reduce bone loss [105]. Not surprisingly, such an effect was not found in a cohort of patients with Crohn’s disease [106]. Therefore, the above-mentioned cytokines may be related to each other in combined fashion [88,92,93,94,96,101,102,103].

Hypogonadism also affects osteoclast activation [73]. A study using osteoclast-specific androgen receptor knockout reported that testosterone likely has both direct effects on the bone via signaling through the androgen receptor in osteoblasts and osteocytes as well as indirect effects on the bone via aromatase activity in osteoblasts [73]. Chronic liver disease is often associated with a change in the metabolism of estrogens along with a decrease in the degradation of estrogen metabolites [107]. Since these estrogens are weak, they are unable to compensate for the estrogen deficiency associated with menopause in women or protect men with chronic liver disease from bone diseases [16,108]. Eventually, hypogonadism causes increased bone turnover [10].

### 4.3. Gut-Liver-Bone Axis

Recently, alterations in gut microbiota (dysbiosis) have been recognized as a possible mechanism of complications in patients with liver cirrhosis [24,109]. The environment, diet, drugs and disease affect the microbiota composition [110] and lead to dysbiosis, an altered microbial community that contributes to disease [27,110,111].

**Figure 3:**
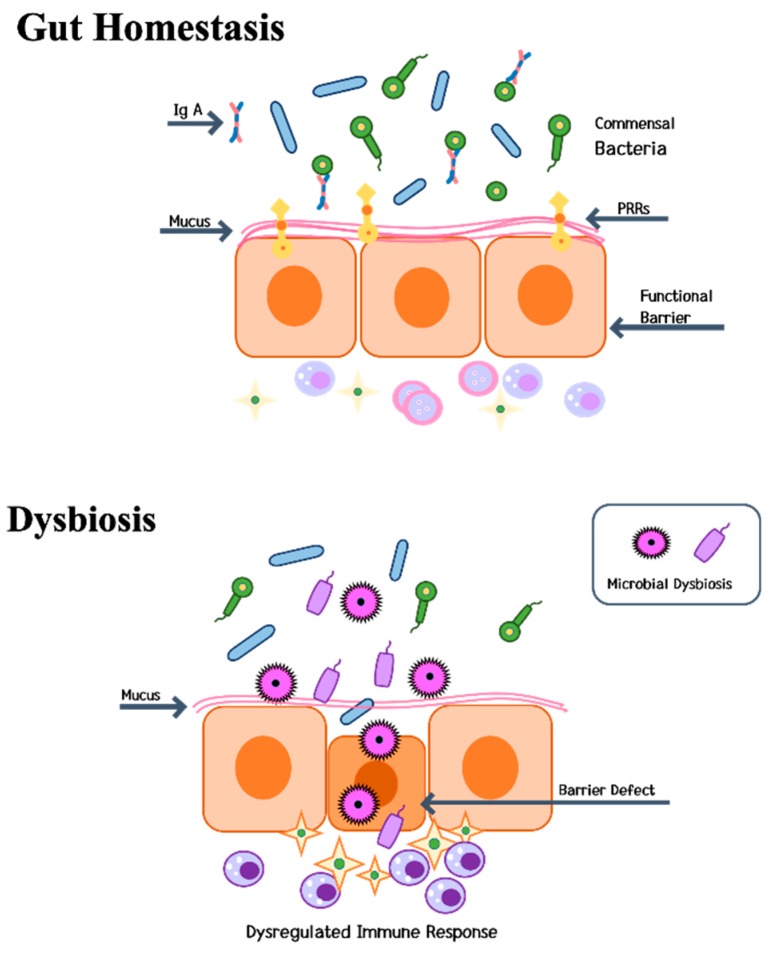
Dysbiosis and gut homeostasis. Dysbiosis has been linked to increased intestinal permeability and a leaky gut. When the balance of the protective bacteria in the intestine collapses and these bacteria are replaced with harmful bacteria, the permeability of the intestinal mucosa is abnormally altered, causing the disappearance of the protective barrier and the induction of inflammation. IgA: immunoglobulin A; PRRs: pattern recognition receptors.

Dysbiosis is associated with increased intestinal permeability, which is known as the “leaky gut syndrome” (Figure 3) [112,113,114,115,116]. Since the liver is the organ in closest contact with the intestinal tract, it is exposed to a substantial number of bacterial components and metabolites [24]. Alterations in host bacteria increase the colonization of pathogenic bacteria, leading to persistent inflammation. In 2007, Cani et al. discovered that gut microbes are involved in low-grade inflammation by activating toll-like receptor (TLR) signaling pathways [114]. Constituents of gram-negative bacteria lipopolysaccharides (LPS) circulate at a very low level in the blood and trigger low-grade inflammation [115,116].

A clinical association between inflammatory conditions and bone loss has long been established [117], and the importance of the immune system in regulating bone remodeling was recognized three decades ago [27,118]. Short-chain fatty acids (SCFAs) produced by gut microbiota are the most widely investigated in relation to regulation of inflammation and immune system. Beyond SCFAs, there are other metabolites, such as indole derivatives and polyamines from digested food that have important immunomodulatory function including T cell differentiation [29].

The relationship between microbiota and bone was first recognized in 2012 [119]. Sjögren et al., in their seminal paper, demonstrated that the gut microbiota modulates the development of immune status and is a major regulator of bone mass in mice. Germ-free (GF) mice exhibited increased bone mass associated with a reduced number of osteoclasts per bone surface compared with conventionally raised mice. Colonization of GF mice with normal gut microbiota could normalize bone mass. Furthermore, GF mice had decreased frequency of CD4^+^ T cells and CD11b^+^/GR 1 osteoclast precursor cells in the bone marrow, which could be normalized by colonization. In summary, gut microbiota regulated bone mass in mice, and there is evidence for a mechanism involving altered immune status in bone affecting osteoclast-mediated bone resorption [119]. Mechanistic studies have shown that microbiota regulate bone formation by altering the production of IGF-1, an important regulator of bone remodeling [120]. Recognizing this, Ohlsson and Sjögren coined the term “osteomicrobiology” suggesting the importance of microbiota in bone immunology and bone health [121].

Maintaining gut tightness is critical in the context of bone formation because osteoclastogenic cytokines are produced by immune cells that reside in intestinal sub-epithelial compartments of the intestine [24]. Any change in gut permeability (“leaky-gut syndrome”) is thus likely to elevate the levels of osteoclastogenic cytokines and influence bone density [122]. Disorders of intestinal microbiota can increase permeability of the intestinal cell, which introduces more LPS into the circulation system. LPS can upregulate inflammatory mediators such as IL-1, cyclooxygenase (COX)-2 and TNF in the bone metaphyseal region.

Interestingly, Li et al. [123] showed that germ-free mice, when chemically castrated with leuprolide, do not lose their bone—a finding that unequivocally establishes the role of gut microbiota in mediating hypogonadal bone loss. Additionally, and not unexpectedly, probiotics reversed hypogonadal osteopenia in sex steroid–deficient mice by preventing the disruption of gut barrier function and dampening cytokine-induced inflammation [123]. The observation that osteoprotection due to absence of microbiota is reversed by reintroduction of flora into germ-free mice proved the fundamental role of gut microbiota in regulating skeletal integrity [105]. Molecules including TNFα, IL-1, IL-6, IL-17, RANKL and OPG drive osteoclastic bone resorption and decrease bone mass [123]. Estrogen serves to dampen this proinflammatory cascade through the gut via several mechanisms [107,108,123]. Estrogen augments gap junction and cell-to-cell contacts, thus preventing the microbiota from inducing inflammation. It also directly suppresses pro-inflammatory T cell production and, indirectly, lowers follicle-stimulating hormone (FSH) levels, thus attenuating FSH-induced TNFα production [123]. Probiotics likely recapitulate many of the anti-inflammatory actions of endogenous estrogen, such as augmenting epithelial cell contacts [105,123].

In other seminal papers published in 2016, Blanton et al. performed transplanting microbiota from six- and 18-month old healthy or undernourished Malawian donors into young germ-free mice fed a Malawian diet [124]. Colonization of germ-free mice with stool samples from malnourished children resulted in stunted body growth and shorter bones, whereas germ-free mice on the same diet given “mature” microbiomes of healthy children underwent normal body growth. Co-housing mice shortly after receiving microbiota from healthy or severely stunted/underweight infants demonstrated that invasion of age-/growth-discriminatory taxa from “healthy” to “severely stunted” cagemates’ microbiota ameliorates growth faltering. Adding two invasive species, *Ruminococcus gnavus* and *Clostridium symbiosum*, to the “severely stunted” microbiota also ameliorated growth and metabolic abnormalities [124,125].

In infant mammals, chronic under-nutrition results in growth hormone resistance and stunting. In mice, Schwarzer et al. showed that strains of *Lactobacillus plantarum* in the gut microbiota sustained growth hormone activity via signaling pathways (IGF-1) in the liver, thus overcoming growth hormone resistance [126]. These two studies [124,126] reveal that changes in the microbiota independently regulate bone growth and bone mass acquisition [122,125].

In another important study, Yan et al. [127] comprehensively evaluated the bone phenotype of both germ-free mice colonized with conventional flora and specific pathogen-free mice treated with antibiotics. They demonstrated that microbiota promote both bone formation and resorption with the net effect on bone dependent on duration of colonization. They further suggest that the effects of microbiota on bone are mediated by the induction of systemic IGF-1, possibly by SCFA [128].

There is no simple explanation for the effects of gut microbiota on bone. Several mechanisms which encompass immune mediated (including Th1, Th17 and Treg), growth factor and hormone mediated (including IGF-1, serotonin and glucagon-like peptide-1 (GLP-1)) and nutrition mediated mechanisms (including calcium and vitamins) have been proposed to explain how the gut microbiome might affect bone at a distance. [26,29,121,122,126,129,130,131,132,133].

## 5. Special Clinical Considerations

### 5.1. Bone Diseases in Patients with a Liver Transplant

As mentioned above, bone diseases are common complications in patients with chronic liver disease [5,6,7,8,9,10]. The problem is more critical in transplant recipients since bone loss is accelerated during the period immediately after LT, leading to a greater incidence of fractures [18,134]. Post-transplantation worsening of bone disease are seen frequently in patients with pre-transplantation bone disease [35,42,135,136,137,138]. Osteoporosis is a serious complication that occurs after LT, with post-operative fractures observed in up to 35% of patients [11,136,137]. Although its pathogenesis is multi-factorial, preexisting bone disease is a major risk factor in patients with chronic liver disease [2,136,138,139] and post-transplant medication may be another factor [136]. In addition, sudden activation of bone remodeling may induce the progression of bone diseases [140,141,142,143]. Specifically, marked bone loss of the proximal femur at Ward’s triangle was observed compared with the spine [144,145].

Two main phases of bone loss after LT have been identified: early and late post-transplantation periods [146]. Before LT, bone remodeling is in a low bone turnover state [50]. Shortly after LT, during the first three to six months, there is a significant and quantitatively substantial increase in bone turnover which is supported by histomorphometric data [142,143] and an early increase in the levels of biochemical markers of bone resorption that exceeds the levels of bone formation markers [147]. In this period, a normalization of mineral metabolism disorders is observed [18]. Gonadal function and 25-hydroxyvitamin D levels are normalized or significantly increased at three months [142,145]. In this early phase after LT, rapid bone loss and a high rate of fractures are associated with high bone turnover, due to the negative effect of the imbalance between bone resorption and formation [142]. In addition, side effects of immunosuppressive therapy, which depend on the doses of glucocorticoids combined with cyclosporine or tacrolimus, contribute to this state in the early post-transplantation phase [148,149].

The second phase, which generally begins six months after LT, is characterized by an improvement in the histological parameters and the biochemical markers of bone formation [147,149]. In a study comparing bone histology before and after LT, this phase of bone turnover exerted a positive effect on bone turnover [142,143]. Factors involved in bone turnover in this second phase after LT are the normalization of liver function and the gradual reduction in glucocorticoids doses, which decrease bone resorption, as well as a secondary hyperparathyroidism related to cyclosporine and tacrolimus administration [18,136].

Notably, the immunosuppressive drugs used after LT are also an important factor [136,150,151,152]. Specifically, glucocorticoids play a pivotal role [151,152]. These compounds stimulate bone resorption and reduce bone formation by inhibiting osteoblast proliferation and differentiation and by promoting apoptosis of osteoblasts and osteocytes [1,151,152]. Generally, the loss of bone mass caused by glucocorticoids begins early and is more pronounced during the first 6–12 months, particularly at the level of the trabecular bone (the vertebrae) [1]. Furthermore, if other risk factors are present, the negative effect of glucocorticoids on bone is further accelerated [152].

Collectively, bone disease that rapidly deteriorates in the early phase of LT is caused by both the rapid bone turnover rate and subsequent use of immunosuppressive agents. A low BMD before LT in patients with chronic liver disease is the greatest risk factor for the deterioration of early bone disease after transplantation. Thus, BMD should be measured before LT as an appropriate form of preparation [35].

### 5.2. Association between Chronic Hepatitis B Virus Infection Treatment and Bone Diseases

Tenofovir disoproxil fumarate (TDF), a commonly used first-line treatment for hepatitis B virus (HBV) infection, is a nucleotide analog that was approved in 2001 as a treatment for human immunodeficiency virus (HIV) infection and more recently for chronic HBV infection [37,38,39,153,154]. TDF is a prodrug that is absorbed from the gut and is released as tenofovir [153]. Loss of BMD is one of the major side effects that have been observed since its development [155]. The relationship between bone diseases (osteoporosis and fracture) and TDF has been well documented in several clinical studies of patients co-infected with HIV and treated with HBV therapy [156]. The TDF-induced decrease in BMD is mainly observed in the hip and spine [157]. TDF usage is identified as an independent risk factor in addition to age, smoking and weight [158]. Most studies have compared patients treated with TDF with controls, but the severity of liver disease has not been considered [157].

Although TDF is generally considered safe and well tolerated [159], concerns regarding the potential long-term side effects associated with prolonged use have been noted [160]. These concerns focus primarily on the reported adverse effect on BMD [161]. Observational data from the veterans’ HIV-infected cohort revealed that cumulative TDF exposure was an independent predictor of increased risk of osteoporotic fracture (hazard ratio 1.08, 95% CI 1.02–1.15) [162]. In this study, the direct effect of TDF treatment on BMD was not apparent due to HIV infection. Gill et al. compared the chronic hepatitis B group with the control group and postulated that the use of TDF may be associated with BMD loss, consistent with previous reports on changes in BMD in HIV-infected cohorts [161]. A decrease in BMD in TDF-treated patients with chronic hepatitis B was only observed in the hip. A recent study showed a plateau in the decrease in hip BMD after 72 weeks of TDF therapy and thus this therapy-associated decrease in BMD may not be a progressive phenomenon [163].

While the pathogenesis remains unclear, several studies have confirmed a negative effect of nucleoside reverse transcriptase inhibitor (NRTI) implicated in mitochondrial dysfunction [164]. Since TDF contains phosphonate, this formulation might conceivably be selectively taken up by osteoclasts in bone through a mechanism similar to bisphosphonates, ultimately leading to cellular stress [153]. The resulting cellular stress would likely inhibit cellular DNA synthesis and gene expression. The reduction in the expression of an osteoclast-related gene that is involved in inducing osteoblast activity would ultimately result in a loss of BMD [153,158]. The loss of BMD due to TDF exposure might also be associated with TDF-induced renal dysfunction, particularly renal proximal tubule dysfunction [165,166]. The failure of renal proximal tubular cells to reabsorb filtered bicarbonate from the urine would result in urinary bicarbonate wasting and subsequent acidemia, known as Fanconi’s syndrome [167]. Therefore, TDF-associated BMD loss may be an outcome of renal dysfunction [153]. Overall, TDF may exert a direct or indirect effect on bone diseases, and while imbalance in bone remodeling is the most likely explanation, additional research is needed [166].

### 5.3. Association of Sarcopenia with Bone Diseases in Patients with Chronic Liver Disease

Sarcopenia is a major risk factor and a major component of aging-related illnesses [3,168]. This condition is the same but more serious in patients with chronic liver disease.

The liver plays an important role in controlling nutritional status and energy balance [41,169]. As a consequence of metabolic impairment associated with liver cirrhosis, protein and amino acid metabolism is likely to be altered, and a continuous breakdown of adipose and muscle tissue components that exceeds oral feeding can occur [21,170]. This condition is associated with a lower body mass index (BMI), a risk factor for osteoporosis [41]. In a recent study that investigated bisphosphonate-mediated improvement in BMD in patients with cirrhosis, the odds ratio for osteoporosis related to BMI was 0.847 (95% CI 0.764–0.94) [171]. In fact, sarcopenia itself is more strongly correlated with osteoporosis than BMI, as observed in elderly individuals who may present with a normal body weight and BMI, but have significant depletion in muscle mass [172]. These individuals may also present with deteriorated bone that is undetectable by assessing body weight alone [173,174,175,176].

Sarcopenia must be screened and treated in patients with chronic liver disease [41], particularly in patients with liver cirrhosis. However, muscle mass is influenced by various factors such as strength exercise and specific hormones (e.g., androgens and β-adrenergic agonist) [177], and thus it is difficult to evaluate accurately [178]. Furthermore, the reference muscle range is not firmly established in patients with chronic liver disease [41]. Nevertheless, psoas muscle and possibly para-spinal and abdominal wall muscles can be considered core skeletal muscles that are relatively independent of activity and water retention. This muscle area is then normalized to height to calculate the skeletal muscle index (cm^2^/m^2^) [41]. Cutoff values defining sarcopenia derived from patients with cirrhosis on the LT list and based on clinical outcomes have recently been suggested (50 cm^2^/m^2^ for men and 39 cm^2^/m^2^ for women) [22]. However, this evaluation method has practical limitations, including radiation exposure due to CT scanning, and the proposed cut-off value is used for patients with cirrhosis among liver transplant recipients. Additional studies are needed.

### 5.4. Association of Chronic Liver Disease with Avascular Necrosis of the Hip

Avascular necrosis (AVN) is a type of bone necrosis caused by insufficient blood flow that predominantly affects several areas of the bone, most commonly the femoral head, in patients with poor circulation [23].

Liver cirrhosis leads to severe disturbances in systemic circulation [179,180], immune response [181,182], and coagulation system [183,184]. In fact, the pathogenesis of cirrhosis and AVN share some common features, although the exact mechanism is not known [185]. Hung et al. [185] reported the time to hospitalization for osteonecrosis of the femoral head (OFN) in patients with cirrhosis (*n* = 40,769) and an age-matched cohort of hospitalized patients with a high prevalence of conditions that predispose them to AVN for three-year follow-up. The proportion of patients with cirrhosis and comparison patients who experienced OFN after three-year follow-up were 0.8% and 0.3%, respectively (*p*-value < 0.001) [185]. Another study hypothesized that potential confounding factors underestimate the strength of the association between cirrhosis and AVN [180]. Deleuran et al. identified confounding conditions predisposing an individual to AVN, such as diabetes, HIV infection, myeloproliferative disease, hemoglobinopathy, chronic renal failure and solid organ transplantation. Additionally, smoking-related conditions (chronic obstructive pulmonary disease), corticosteroids treatment (autoimmune hepatitis, rheumatoid arthritis and connective tissue disease) and alcohol were also considered confounding factors [180]. Chronic inflammation may be more important for the development of AVN in patients with chronic liver disease [182,186]. Interleukin-33, a T-lymphocyte activator has been linked to both cirrhosis and AVN [187]. In summary, although AVN is a rare condition even in patients with cirrhosis, cirrhosis is a strong risk factor for AVN requiring total hip arthroplasty [180].

## 6. Management

As mentioned above, a low BMD in patients with chronic liver disease significantly affects their quality of life of patients. Nutritional, hormonal, metabolic, genetic and inflammatory factors play important roles in the pathogenesis of osteoporosis in patients with chronic liver disease [41].

### 6.1. Approaches to Risk Management and Overall Nutritional Status

Optimal therapeutic intervention would improve liver function, diminish inhibitory signals (e.g., bilirubin) and increase stimulating signals for bone formation (e.g., IGF-1) that originate from the liver [30]. Treatment of bone disease in patients with chronic liver disease should be approached in various ways, including the improvement of liver disease and elimination of risk factors, rather than a single treatment. Thus, treatment of bone diseases in patients with chronic liver disease begins with removing contributing risk factors. Factors [6] contributing to bone loss should be minimized, such as encouraging alcohol abstinence and smoking cessation. Corticosteroids should also be minimized whenever possible (Table 3) [18].

Although no clear anti-fracture effect of nutritional support has been established, nutritional aspects should be considered during therapy [41]. Balanced nutritional support should be prescribed since patients with advanced liver disease frequently have little appetite and are malnourished [41].

Specifically, caloric intake recommendation is 30–50 kcal/kg/day for restoring or maintaining nutritional status and enhancing liver regeneration [188]. Protein intake recommendation is 1.0–1.8 g/kg body weight/day depending on the severity of malnutrition [188]. For carbohydrates, the recommendation is 45–75% of total caloric intake by (4–6 times) small meals per day [188]. For lipids, 20–30% of caloric intake is recommended [188]. Branched chain amino acids (BCAAs) include leucine, isoleucine and valine, which cannot be synthesized in the body and must be obtained through diet [189]. There have been multiple research studies on the use of BCAAs for nutritional support [190]. Collectively, early identification and treatment of malnutrition has the potential to lead to better disease outcomes and prevent complications such as osteoporosis [188].

### 6.2. Pharmacological Therapies for Bone Diseases

The number of therapeutic options has increased in recent years [1,5,19,46]. Various guidelines from osteoporosis societies on pharmacological therapies are designed for the treatment [1,10,15,19] of common osteoporosis, and are not specific for patients with chronic liver disease. Nevertheless, until the development of chronic liver disease-specific guidelines, pharmacological therapies for bone diseases in patients with chronic liver disease should be based on those common osteoporosis guidelines in addition to the alleviation of inflammation related to chronic liver disease.

### 6.3. Calcium and Vitamin D Supplementation

Most studies recommend calcium and vitamin D supplementation [41]. Total calcium intake should achieve a daily value of 1000 to 1500 mg/day, according to age and other factors [1,10,15,19]. The supplement that is most widely consumed by patients is calcium carbonate, which must be ingested along with foods to increase absorption [1,5,10,15,19]. Calcium citrate is more suitable for patients with achlorhydria or other conditions that potentially impair gastrointestinal absorption [15,19]. Calcium tablets should never be ingested with fluoroquinolones, tetracycline, bisphosphonates, phenytoin, or levothyroxine because the supplements impair the absorption of these drugs [10].

Oral 25-hydroxy vitamin D supplements are typically prescribed at a dose of 400–800 IU/d or 260 µg every 2 weeks [1,15,19]. Since calcitriol (1,25-dihydroxy cholecalciferol) is the final active metabolite of vitamin D, it appears to be a better treatment for these patients. Calcitriol is usually prescribed as a daily oral dose of 800 U, but can also be taken at a weekly dose of 5000 U [12]. In a clinical trial in which calcitriol (0.5 mg twice per day) was administered to 38 patients with cirrhosis for 12 months, the supplement was the only factor that significantly correlated with an increase in BMD [191].

Although calcium and vitamin D are widely prescribed to patients with osteoporosis [1,10,15,19], compliance might be a problem. The number of elderly non-cirrhotic patients who reported more than one year of continuous use was less than 50% [192]. However, several studies have shown that the continued use of these supplements is consistent with their positive effects on bone loss, suggesting that continuous use is recommended in patients with chronic liver disease [193].

### 6.4. Pharmacological Therapies that Increase Bone Formation

Intermittent administration of parathyroid hormone has been shown to stimulate bone formation to a larger extent than bone resorption [1,10,15,19]. This effect is achieved through the activation of osteoblasts by inhibiting sclerostin, which prevents Wnt signaling [78]. However, evidence supporting this effect on patients with chronic liver disease is not available.

Sodium fluoride is also known to increase lumbar spine bone mass in patients with osteoporosis [194]. After two years of treatment with fluoride and etidronate, a subtle increase was found in vertebral BMD in the PBC patients [195]. Additional studies are also needed to confirm the efficacy and safety of sodium fluoride for patients with cirrhosis [10,12]. The administration of IGF-1 appears to be beneficial in rats, but no studies of IGF-1 use in humans exist [65,67,69,73,196].

### 6.5. Pharmacological Therapies that Decrease Bone Resorption

Inhibition of osteoclast activation and bone resorption to achieve a positive balance in bone is the core of anti-resorptive treatments [1,10,15,19]. These treatments include bisphosphonates, selective estrogen receptor modulators (SERMs) and RANKL inhibitors [197].

Bisphosphonate, one of the potent inhibitors of bone resorption, is the most frequently prescribed drug worldwide [3]. Bisphosphonate is a derivative of pyrophosphate, which is very stable, and most of it is absorbed into the BRU and enters the exposed osteoclast and inhibits its activation [198,199]. Currently available bisphosphonate preparations include alendronate, risedronate, ibandronate, pamidronate and zoledronate [1,10,15,19]. Of these formulations, ibandronate, pamidronate and zoledronate have also been administered as IV preparations [200].

Alendronate exerts a positive effect on BMD in patients with LT [201]. The BMD of the lumbar spine in the alendronate group increased by 5.1% ± 3.9% at 12 months and was significantly higher than the non-alendronate group (0.4% ± 4.2%) [201]. Dodidou et al. evaluated 21 patients treated with infusions of pamidronate (30 mg) every three months after LT combined with vitamin D and calcium supplements. The authors reported a significant increase in the lumbar spine and femoral neck BMD in patients who received pamidronate, which persisted during the second year of treatment [202,203]. In a recent study, Bansal et al. showed a clear improvement in BMD of patients with non-cholestatic cirrhosis who were administered ibandronate [171]. According to Bodingbauer et al., the anti-resorptive action of zoledronate observed after six months results in beneficial effects on bone matrix mineralization, likely contributing to the significant decrease in fracture incidence observed in these patients at two years after LT [204].

Although large-scale studies are needed, studies investigating patients with chronic liver disease, such as transplant recipients and patients with PBC [205], revealed some beneficial effects of bisphosphonate. It is considered the most powerful drug for patients with chronic liver disease [5,12,18]. Limitations associated with bisphosphonate use may be observed due to side effects and compliance [206]. A typical side effect is esophagitis, which can occur when patients are taking oral preparations [206]. The use of the IV form of bisphosphonates, such as zoledronate, which is given once a year, might improve compliance [207].

SERMs have also been administered as anti-resorptive therapies [1,10,15,19]. Raloxifene is a second-generation SERM [10,208] that functions as an estrogenic agonist in bone by decreasing bone resorption and bone turnover, thus increasing the BMD [14]. Raloxifene has also been used as a weaker anti-resorptive therapy for higher-risk patients during a “bisphosphonate holiday” [19]. It has been used to treat osteoporosis in patients without liver disease, but has not yet been tested in patients with cirrhosis [10]. A previous study was performed in nine postmenopausal women with PBC, suggesting a possible beneficial effect on lumbar spine BMD [209,210].

Hormone therapy was also expected to increase the BMD in patients with chronic liver disease [12]. However, despite the positive results, hormone therapy is not considered an appropriate treatment, due to concerns about malignancy and the availability of other efficacious non-hormonal agents with fewer side effects [12,211].

Recently, the American Association of Clinical Endocrinologists/American College of Endocrinology (AACE/ACE) recommended denosumab as a first-line therapy for patients at high risk of fracture and for patients who are unable to use oral therapy [19]. Denosumab is a fully humanized monoclonal antibody to RANKL [212]. The therapeutic effect of denosumab is based on its capability to inhibit osteoclast differentiation [213]. Although the RANKL pathway has no recognized effect on the liver, drug-induced liver injury cases have been reported, though very rare [213]. Denosumab is expected to play a major role in the treatment of bone diseases in patients with chronic liver disease, along with bisphosphonate [41].

### 6.6. Therapies Targeting Gut Dysbiosis

As mentioned above, chronic inflammation induces bone resorption by activating immune cells [29,122]. This change is particularly related to the gut microbiota [122]. The gut microbiota of patients with chronic liver disease is different from healthy individuals and is known to influence the development and function of the host immune system [214]. Therefore, the relationship between dysbiosis and bone diseases is worth exploring. An association between a low intestinal bacteria count and decreased BMD has been reported in several clinical trials [214]. Several studies have reported an association of dysbiosis with chronic inflammation, which may in turn result in an association with bone diseases [129,132,215,216]. Various liver diseases, such as alcoholic liver disease, non-alcoholic fatty liver disease and PSC, are associated with altered microbiota [24,109], which can affect immunomodulation [29,122]. Probiotic administration reduces the expression of several pro-inflammatory and osteolytic cytokines, such as TNF-α and IL-1β [217]. Many complications of chronic liver disease are anticipated to be treated by strategies that manipulate the microbiota [114]. However, until date, evidence for the role of gut microbiota in bone health in humans remain inadequate [26,129,131].

## 7. Conclusions

Osteoporosis is a frequently observed complication in patients with chronic liver disease, particularly liver cirrhosis and cholestatic liver diseases. In addition, the problem is critical in liver transplant recipients. Few studies have evaluated bone diseases in patients with more frequently observed chronic liver disease, such as chronic viral hepatitis, non-alcoholic fatty liver disease and alcoholic liver disease.

In the last few decades, many developments have improved our knowledge of the pathogenic mechanisms and management of osteoporosis. However, many issues remain to be solved in patients with chronic liver disease. Although nutritional, hormonal, metabolic and genetic factors may be important contributors, inflammation may be the most persistent and probable cause of bone diseases in patients with chronic liver disease. Recently, studies regarding the effect of chronic inflammation on bone diseases, including those related to dysbiosis, have been conducted.

Due to the increased life expectancy and advances in management of chronic liver disease, the importance of managing osteoporosis and other bone diseases in patients with chronic liver disease is expected to increase. Consequently, guidelines specific for bone diseases in patients with chronic liver disease need to be established in the near future.

## Figures and Tables

**Table 1:** Guidelines for bone diseases in patients with chronic liver disease.

	Guidelines or Guidance	Bone Diseases Related to Liver Disease
EASL ^1^	2018 Clinical Practice Guidelines: Nutrition in chronic liver disease [41]	Nutritional treatment options in cirrhotic patients with bone diseases
EASL ^1^	2017 Clinical Practice Guidelines: Management of hepatitis B virus infection [38]	Indications for selecting entecavir or tenofovir alafenamide over tenofovir disoproxil fumarate
EASL ^1^	2017 Clinical Practice Guidelines: Primary biliary cholangitis [20]	Management of complications: osteoporosis
EASL ^1^	2016 Clinical Practice Guidelines: Liver transplantation [2]	Bone disease screening and management
EASL ^1^	2015 Clinical Practice Guidelines: Autoimmune hepatitis [36]	Osteopenia/osteoporosis screening and management
AASLD ^2^	2018 Primary Biliary Cholangitis: Practice guidance [40]	Complication related to chronic cholestasis: osteoporosis/osteopenia management
AASLD ^2^	2018 Hepatitis B Guidance: Update on prevention, diagnosis and treatment of chronic hepatitis B [39]	Tenofovir disoproxil fumarate-associated bone disease
AASLD ^2^	2013 Evaluation for Liver Transplantation in Adults: Practice guideline [35]	Bone densitometry as part of transplant evaluation and treatment of osteoporosis initiated prior to liver transplantation
AASLD ^2^	2012 Practice guidelines by AASLD and AST ^3^: Long-term management of the successful adult liver transplant [34]	Bone mineral density follow up and management
AASLD ^2^	2010 Diagnosis and Management of Primary Sclerosing Cholangitis [33]	Evaluation and management of bone disease in PBC patients
APASL **^4^**	2015 Clinical Practice Guidelines on the Management of Hepatitis B [37]	Decline in the bone mineral density in tenofovir disoproxil fumarate treatment
Collier et al.	Guidelines on the Management of Osteoporosis Associated with Chronic Liver Disease [9]	Review of the assessment and diagnosis of osteoporosis, the therapeutic agents available, and the way in which they can be used in patients with chronic liver disease to prevent osteoporosis

1. EASL, European Association for the Study of the Liver; 2. AASLD, American Association for the Study of Liver Diseases; 3. AST, American Society of Transplantation; 4. APASL, Asian-Pacific Association for the Study of the Liver.

**Table 2:** Predominant changes in bone in various liver disease.

Predominant Changes in Bone Cell Activity in Various Liver Disease	
**Increased Resorption**	Viral hepatitis
	Transplantation
	Corticosteroid therapy
**Decreased Formation**	Cholestatic liver diseases
	Iron and copper overload

**Table 3:** Risk factors for a low bone mass.

Risk Factors for a Low Bone Mass and Fragility Fractures
Advanced age
Osteoporosis
Previous fragility fracture
Menopause
Male hypogonadism
Immobilization or physical inactivity
Excess alcohol intake
Low body mass index
Chronic cholestasis
End-stage liver disease
Long-term corticosteroid therapy ( >5 mg for more than three months)
Immunosuppressive agents
